# Study on Multi-GNSS Precise Point Positioning Performance with Adverse Effects of Satellite Signals on Android Smartphone

**DOI:** 10.3390/s20226447

**Published:** 2020-11-11

**Authors:** Hongyu Zhu, Linyuan Xia, Dongjin Wu, Jingchao Xia, Qianxia Li

**Affiliations:** 1 School of Geography and Planning, Sun Yat-Sen University, Guangzhou 510000, China; zhuhy35@mail2.sysu.edu.cn (H.Z.); wudj3@mail.sysu.edu.cn (D.W.); liqianx2@mail2.sysu.edu.cn (Q.L.); 2 School of Civil Engineering, Guangzhou University, Guangzhou 510000, China; jcxia@gzhu.edu.cn

**Keywords:** Android smartphone, precise point positioning, multi-GNSS, stochastic model, robust Kalman filter, gradual accumulation of phase error

## Abstract

The emergence of dual frequency global navigation satellite system (GNSS) chip actively promotes the progress of precise point positioning (PPP) technology in Android smartphones. However, some characteristics of GNSS signals on current smartphones still adversely affect the positioning accuracy of multi-GNSS PPP. In order to reduce the adverse effects on positioning, this paper takes Huawei Mate30 as the experimental object and presents the analysis of multi-GNSS observations from the aspects of carrier-to-noise ratio, cycle slip, gradual accumulation of phase error, and pseudorange residual. Accordingly, we establish a multi-GNSS PPP mathematical model that is more suitable for GNSS observations from a smartphone. The stochastic model is composed of GNSS step function variances depending on carrier-to-noise ratio, and the robust Kalman filter is applied to parameter estimation. The multi-GNSS experimental results show that the proposed PPP method can significantly reduce the effect of poor satellite signal quality on positioning accuracy. Compared with the conventional PPP model, the root mean square (RMS) of GPS/BeiDou (BDS)/GLONASS static PPP horizontal and vertical errors in the initial 10 min decreased by 23.71% and 62.06%, respectively, and the horizontal positioning accuracy reached 10 cm within 100 min. Meanwhile, the kinematic PPP maximum three-dimensional positioning error of GPS/BDS/GLONASS decreased from 16.543 to 10.317 m.

## 1. Introduction

At the May 2016 Google I/O developer conference, it was announced that general developers would be provided with the raw global navigation satellite system (GNSS) measurements of smartphones and tablets with Android N (“Nougat” = version 7) version operating system [1], which enables the raw GNSS observations of smartphone to output directly in RENIX format with application (APP). This makes it extremely convenient to obtain the GNSS pseudorange, doppler, signal strength, and carrier phase measurements from an Android smartphone for postprocessing [2]. Moreover, in September 2017, Broadcom announced the world’s first dual frequency GNSS chip GCM47755 [3], and other producers quickly followed suit with new dual frequency chips. Since then, the GNSS positioning method of smartphones has evolved from single frequency to dual frequency, which provides more ideas for smartphones to achieve high-precision positioning.

In fact, prior to the Google I/O developer conference, Humphreys et al. [4] had discussed the feasibility of centimeter-level positioning via the smartphone’s antenna and GNSS chip. The feasibility study showed that the smartphone pseudorange measurements are of high quality and conform to expected conventions, but the carrier phase measurements suffer from five anomalies. One of the anomalies that cannot be remedied is an error in the phase measurement that appears to grow linearly with time. Nevertheless, the authors still believe that the smartphone appears fully capable of supporting centimeter-level carrier phase differential GNSS positioning. In recent years, as more smartphones are equipped with a dual frequency GNSS chip, in addition to differential positioning method [5,6,7,8,9] the precise point positioning (PPP) method is also used in GNSS high-precision positioning of smartphones [10,11,12]. PPP is a precision absolute point positioning technology based on state space domain correction information with international GNSS service (IGS) products [13,14]. The GPS/Galileo pseudorange and carrier phase observations L1/L5 and E1/E5 of Xiaomi Mi 8 were used in the ionosphere-free combined PPP mathematical model [11], and the experimental results showed that the dual frequency GNSS smartphone is capable of achieving decimeter-level positioning accuracy. Wu et al. [12] compared Xiaomi Mi 8 and geodetic receiver from the perspective of single frequency and dual frequency PPP models, the experimental results showed that the positioning accuracy of dual frequency PPP with ionosphere-free combination on Xiaomi Mi 8 is similar to the geodetic receivers in single frequency PPP mode. However, the scarcity of GPS satellites with L5 band and the poor geometric distribution of Galileo satellites in the Asia–Pacific region make it difficult to conduct dual frequency PPP using smartphone measurements. Wu et al. [12] recorded that Xiaomi Mi 8 received more than four satellites with L5/E5 band for about 13 h in 24-h collection. This makes it difficult to meet the requirement of dual frequency PPP based on GPS/Galileo for a smartphone during the whole day. Therefore, in order to improve the applicability and stability of multi-GNSS PPP on a smartphone, we also need to focus on the quality of B1 and G1 band observations of BeiDou (BDS) and GLONASS. Moreover, some characteristics of GNSS signals can adversely affect positioning performance on a smartphone, which should be further discussed.

To support low power consumption and thus prevent battery drainage, duty cycle power saving techniques are widely used in the smartphone. It intermittently puts the receiving device into a sleep state, especially in static mode the sleep period can be set to the maximum [15]. This function will seriously affect the reception of GNSS carrier phase observations. Wu et al. [12] conducted the duty cycle experiment on the Xiaomi Mi 8, and the rate of cycle slip increased from about 20% to 70% within 30 min after turning on the duty cycle. Li et al. [7] showed that the rates of cycle slip on the Samsung Galaxy S8 and Huawei Honor V8 are greater than 50% and 90% after the duty cycle is turned on. Fortunately, it is possible to switch off the duty cycle mode after Android P (“Pie” = version 9), and [5,6,16] indicated the duty cycling is disabled in the Nexus 9 tablet; even Wanninger et al. [9] showed that the observed carrier phase measurements of the Huawei P30 were continuous without the need to manually stop duty cycling, which seems to be disabled beforehand.

Signal-to-noise ratio (SNR) refers to the ratio between the measured signal intensity and noise intensity at the same time and place in the circuit. Carrier-to-noise power density ratio (*C/N*_0_) is usually used to describe signal noise level in a GNSS receiver, and the *C/N*_0_ value is SNR in 1 Hz width with the unit of dB-Hz. The *C/N*_0_ is commonly affected by atmosphere, multipath, signal receiving antenna, internal circuit, etc. However, the majority of studies showed that the mean *C/N*_0_ value of smartphones is generally lower than that of the geodetic receiver, and the *C/N*_0_ difference between the geodetic receiver and smartphones even exceeds 10 dB-Hz [8,17]. Liu et al. [17] also indicated that the *C/N*_0_ values of the Samsung Galaxy S3 sometimes suddenly and drastically decreased as the elevation increased.

In addition, there are still some special properties such as gradual accumulation of phase errors [4] in GNSS carrier phase observations of smartphones. Chen et al. [10] found the pseudorange and carrier phase observations (in meters) of the geodetic receiver are consistent, but not with the Xiaomi Mi 8 and Huawei Honor 9. This means the difference between pseudorange observations and carrier phase observations (in meters) are not fixed on smartphones. Various drifts of GNSS carrier phase observations, such as the code–phase difference ranged from −50 to 50 m for GPS satellites and −100 to 100 m for GLONASS satellites in Nexus 9 tablet, were also shown in [16]. Moreover, the positioning performance of smartphones is also limited by the poor multipath suppression capability. Lachapelle et al. [18] conducted the PPP experiment using the Huawei Mate20X in low and high multipath environments, even in the static PPP, the RMS of vertical errors in a high multipath environment is over ten times as in a low multipath environment within 60 min. In addition, the positioning accuracy of smartphones with an external high-grade antenna under high multipath can reach that of a smartphone with its own antenna under low multipath.

Unlike the GNSS data observed by the geodetic receiver, the GNSS observations of a current smartphone are always affected by a variety of adverse factors mentioned above due to the hardware performance limitation. In order to reduce the adverse effects on positioning accuracy of multi-GNSS PPP, we first analyze in detail in Section 2 the GNSS signal characteristics, including the duty cycle, carrier-to-noise ratio, carrier phase cycle slip, gradual accumulation of phase errors, and pseudorange residual of smartphones. After that, in Section 3, we establish a multi-GNSS PPP mathematical model for smartphones with a stochastic model that is composed of GPS, BDS, and GLONASS step function variance depending on carrier-to-noise ratio, and the robust Kalman filter is used for parameter estimation. In Section 4, we show the multi-GNSS static and kinematic experiments and results, and the experimental results demonstrate the proposed PPP mathematical model provides higher positioning accuracy and accelerates the convergence of static positioning, compared with conventional PPP method depending on elevation. Lastly, we draw conclusions and add remarks in Section 5.

## 2. GNSS Signal Characteristics

**Figure 1:**
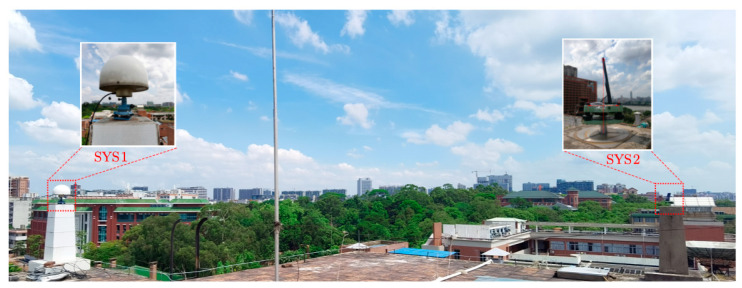
View of the experiment site. Left: global navigation satellite system (GNSS) antenna of the reference station on the reference point SYS1, working all days; right: the Huawei Mate30 is placed on the instrument base above the reference point SYS2 and secured with tape.

This study uses a Huawei Mate30 as the main experimental object, as shown on the right in Figure 1. Huawei Mate30 is an Android smartphone with a Kirin 990 chip developed by Huawei Technologies Co., Ltd., in September 2019, and it supports the L1 and L5 dual bands of GPS, B1 band of BDS, G1 band of GLONASS, and E1 and E5a dual bands of Galileo. Referring to the calibration of the GNSS antenna L1 phase center of the smartphone [9], the antenna phase center is located at the top of the equipment with a small offset to the right, and there no millimeter-accurate phase center exists. Therefore, the Huawei Mate30 is placed on the reference point SYS2. The top point of the smart phone is on the axis that is orthogonal to the plane and crosses the reference point, and the distance between the two points is used as the height of the antenna on the smartphone. On the left in Figure 1, there is a reference station with a CR3-G3 geodetic antenna on the reference point SYS1 northeast of the point SYS2. It should be noted that the Huawei Mate9, Huawei Mate20, and Huawei P30 were also employed in part of the subsequent experiments.

The experiments were conducted in the open area on the roof, and the GNSS observation data of the smartphone were collected and stored by Android app GEO++ RINEX Logger V2.1.3 with RINEX VERSION 3.03, without significant signal obstructions and strong multipath reflectors. The test data were collected over 2 h, and we mainly used the GNSS observations for the first 100 min. The coordinate values of reference points SYS1 and SYS2 were calculated on the NTRF14 in 2020 and treated approximately as an accurate coordinate for comparison in subsequent experiments.

### 2.1. Duty Cycle

The existence of duty cycle affects the continuity of GNSS carrier phase measurements of smartphones. According to the GNSS carrier phase measurements of the four smartphones of Huawei, it is found that the carrier phase measurements of early smartphones, such as the Huawei Mate9, are severely affected by duty cycle. The carrier phase measurements are missing in the logging data of Huawei Mate9 at the beginning after a time period of 3–5 min, but the other measurements such as pseudorange, doppler, and signal strength are still continuously logged. However, with the analysis of the carrier phase measurements of the other three smartphones, our view is consistent with that of [3,9], which hold that some recent smartphones such as the Huawei Mate30 and Huawei P30 are little or not affected by duty cycle even in the standard static positioning mode.

### 2.2. Carrier-to-Noise Ratio

**Figure 2:**
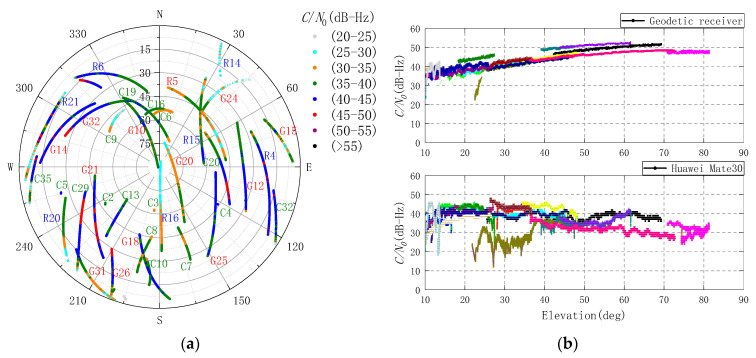
Huawei Mate30 sky plot and the GNSS satellites *C/N*_0_ values of the geodetic receiver and Huawei Mate30. (**a**) Each color corresponds to the *C/N*_0_ range of GNSS L1 band signal. (**b**) The same color on the top and bottom represents the same GNSS satellite. The G, C, and R denote GPS, BeiDou (BDS), and GLONASS, respectively.

The Huawei Mate30 sky plot and *C/N*_0_ range of GPS, BDS, and GLONASS carrier L1 in static data collection are shown in Figure 2a. It should be noted that the number and quality of Galileo observations received by the Huawei Mate30 were lower than expected, and even the number of Galileo satellites could not meet the requirements of standard single point positioning (SPP) for some time. Therefore, the Galileo observations were not used in the experiment. The *C/N*_0_ values of GNSS satellites received by the geodetic receiver and Huawei Mate30 vary with elevation ranges from 10 to 90 degrees, as shown in Figure 2b.

In the Figure 2b, the changing trends for GNSS satellite *C/N*_0_ values of the Huawei Mate30 and geodetic receiver are almost opposite. As the elevation increased, the *C/N*_0_ values of the smartphone were actually decreased. Paziewski et al. [8] showed the *C/N*_0_ values of the Huawei P20 decrease significantly while the elevation is below 30 degrees, which is even below 10 dB-Hz. However, it seems to be improved on the Huawei Mate30; the *C/N*_0_ values of the GNSS satellite are never below 10 dB-Hz in this experiment. Moreover, the *C/N*_0_ values of the Huawei Mate30 do not decrease sharply as shown in [4] when the elevation is above 45 degrees. With the elevation in Figure 2b divided into eight parts on a scale of 10 degrees from low to high, the average difference of *C/N*_0_ values between the geodetic receiver and Huawei Mate30 are −2.92, −0.70, 2.86, 9.90, 13.78, 13.95, 16.85, and 15.23 dB-Hz. The overall average difference is 8.62 dB-Hz, which is approximately in line with prior studies [7,8,19].

### 2.3. Cycle Slip

**Figure 3:**
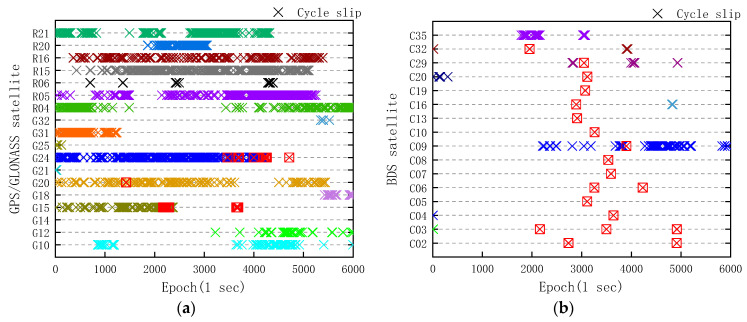
Huawei Mate30 carrier phase cycle slip detection of GNSS L1 band observations. (**a**) GPS and GLONASS; (**b**) BDS. The extra cycle slips of GPS and BDS satellites detected by measurement-based polynomial fitting (MPF) were marked with red boxes.

Carrier phase cycle slip is generally due to a temporary loss-of-lock in the carrier tracking loop of a GNSS receiver, and is an unknown integer number of cycles varying from one to millions [20]. In general, cycle slips are caused by obstructions of satellite signal path, low carrier-to-noise density ratio, or failed receiver software [21]. Wu et al. [12] showed the experiment of carrier phase cycle slip of the Xiaomi Mi 8; even with the duty cycle being switched off, the cycle slip percentage was close to 20%. According to the GNSS analysis app release notes by Google, the state of the “Accumulated Delta Range” can detect the carrier phase reset and loss-of-lock on a smartphone. The cycle slips detected by the Huawei Mate30 itself are shown in Figure 3.

The cycle slip percentage of GPS, GLONASS, and BDS satellites are 1.85%, 4.04%, and 0.18% in 6000 epochs, respectively. However, it is not rigorous for cycle slip detection only depending on the smartphone itself. Therefore, the measurement-based polynomial fitting (MPF) method was used to detect cycle slip again. The basic principle of MPF is to conduct polynomial fitting for the phase observations of several epochs, and the *k*th epoch observation can be expressed as [22]:

(1) $$E\left({\tilde{\varphi }}_{i}\right)=a_{0}+a_{1}k+a_{2}k^{2}+\cdots +a_{m}k^{m}$$

where i=1,2,⋯,n(n>m+1) and $a_{0},a_{1},\cdots ,a_{m}$ are the array coefficients, which are computed by the least squares method. The formal standard deviation (STD) of unit weight is computed as:

(2) $$\sigma =\sqrt{\frac{\left[V^{T}V\right]}{n-m-1}}$$

where V is residual error. The number of extra GPS and BDS cycle slips detected by MPF is 25 and 19, respectively, and we mark them with red boxes in Figure 3. Surprisingly, thousands of extra GLONASS cycle slips are detected by MPF, and it is difficult to mark them in Figure 3a. We conducted extensive experiments at a different time, and it is also occurred in the experiments conducted using the Huawei Mate20 and Huawei P30. Therefore, we believe that the cycle slip phenomenon of GLONASS seems to relate to the GNSS chip in smartphones. From Figure 3 and Figure 2a, it can be found that the cycle slip rarely occurs when the *C/N*_0_ value is above 40 dB-Hz, and it gradually occurs while *C/N*_0_ value changes from 40 to 30 dB-Hz. With the *C/N*_0_ value less than 30 dB-Hz, the number of carrier phase cycle slips increased significantly.

### 2.4. Phase-Code Differences

The gradual accumulation of phase errors cannot be ignored when high-precision positioning is carried on using measurements from a smartphone. The phase–code combination between satellite and receiver in meters is given directly as [8,16]:

(3) $$L_{i}-P_{i}=-2\frac{f_{1}^{2}}{f_{i}^{2}}I-\lambda_{i}N_{i}+B_{L_{i}}^{r}-B_{P_{i}}^{r}-B_{L_{i}}^{s}+B_{P_{i}}^{s}+\varepsilon_{L_{i}}-\varepsilon_{P_{i}}$$

where the subscript i denotes frequency, L denotes raw phase observation scaled to distance, P denotes raw code observation, $f_{i}$ is the frequency of $L_{i}$, I is the ionospheric delay on frequency $L_{1}$, $\lambda_{i}$ is the wavelength of $f_{i}$, $N_{i}$ is the integer ambiguity of $L_{i}$ in cycle, the symbols $B_{P_{i}}^{r}$, $B_{L_{i}}^{r}$, $B_{P_{i}}^{s}$, and $B_{L_{i}}^{s}$ are code and phase hardware delay of receiver and satellite, respectively, and the ε terms are unmodeled errors including code and phase multipath effect and observation random noise. Part of ionospheric delay in Equation (3) can be eliminated with IGS final global ionospheric map (GIM) products, and the difference value $D_{i}$ is written as:

(4) $$D_{i}=L_{i}-P_{i}+2\frac{f_{1}^{2}}{f_{i}^{2}}I=-\lambda_{i}N_{i}+(B_{L_{i}}^{r}-B_{P_{i}}^{r})-(B_{L_{i}}^{s}-B_{P_{i}}^{s})+\varepsilon_{L_{i}}-\varepsilon_{P_{i}}$$

**Figure 4:**
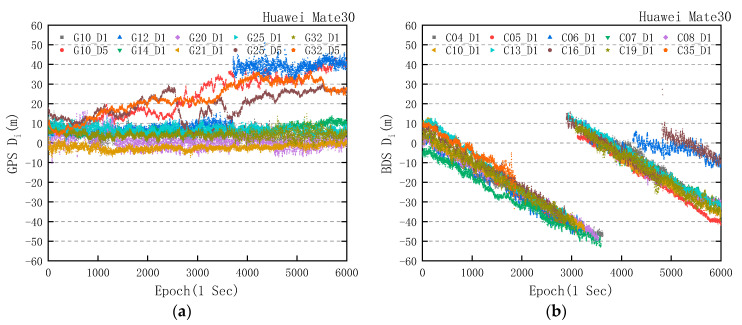

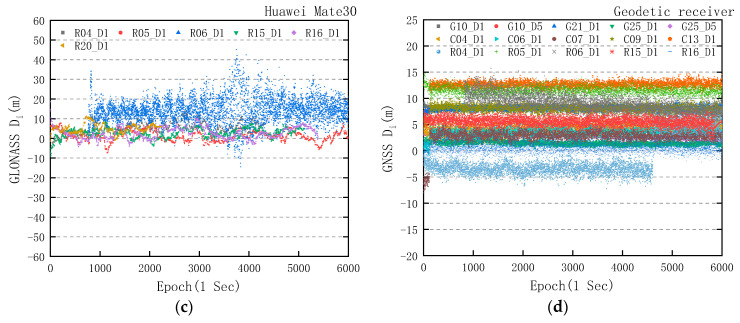
Difference $D_{i}$ of observed GPS, BDS, and GLONASS satellites in static data collected by the Huawei Mate30 and geodetic receiver. (**a**) Huawei Mate30 difference $D_{i}$ of GPS; (**b**) Huawei Mate30 difference $D_{i}$ of BDS; (**c**) Huawei Mate30 difference $D_{i}$ of GLONASS; and (**d**) geodetic receiver difference $D_{i}$ of GPS, BDS, and GLONASS.

The value of $D_{i}$ is mainly affected by integer ambiguity, code and phase hardware delay of receiver and satellite, multipath effect, and measurement noise. Therefore, the $D_{i}$ values always fluctuate within a fixed range, and the trends of $D_{i}$ values of the Huawei Mate30 and geodetic receiver are shown in Figure 4.

From Figure 4d, the trends of $D_{i}$ values of GPS/BDS/GLONASS in the geodetic receiver are in line with the expectation. In Figure 4a, the significant change of $D_{i}$ value mainly concentrates in the L5 band, and the changing trends of $D_{i}$ value of GPS in L1 band are similar to that of the geodetic receiver. This is different from the experimental result of the Huawei P20 in [8], and it indicates that the quality of GPS carrier phase observations of the Huawei Mate30 has been improved. However, in Figure 4b, we clearly found that all of the BDS carrier phase observations in the Huawei Mate30 were affected by the gradual accumulation of phase errors, and the trends correspond to a change that ranged from −1.203 to −1.548 cm/s, with a mean value of −1.401 cm/s. In addition, when the difference value between the carrier phase observations (in meters) and the pseudorange observations reached the threshold of 50 m, the carrier phase observations (in meters) were consistent with the pseudorange by means of cycle slip. In Figure 3b, taking BDS pseudo random noise (PRN) 04 and PRN 16 for example, the extra cycle slips of PRN 04 and PRN 16 were detected in 3640 epochs and 2890 epochs, which was the same as the time when the carrier phase observations (in meters) were consistent with pseudorange in Figure 4b. The phenomenon of cycle slip after reaching the threshold did not happen in other similar experiments [8,16]. For GLONASS in Figure 4c, a large number of cycle slips, except for the R6 satellite, make carrier phase observations and pseudorange observations coincide with each other. However, there seems to be no accumulated deviation from the $D_{i}$ value in GLONASS carrier phase observations.

### 2.5. Pseudorange Residual

**Figure 5:**
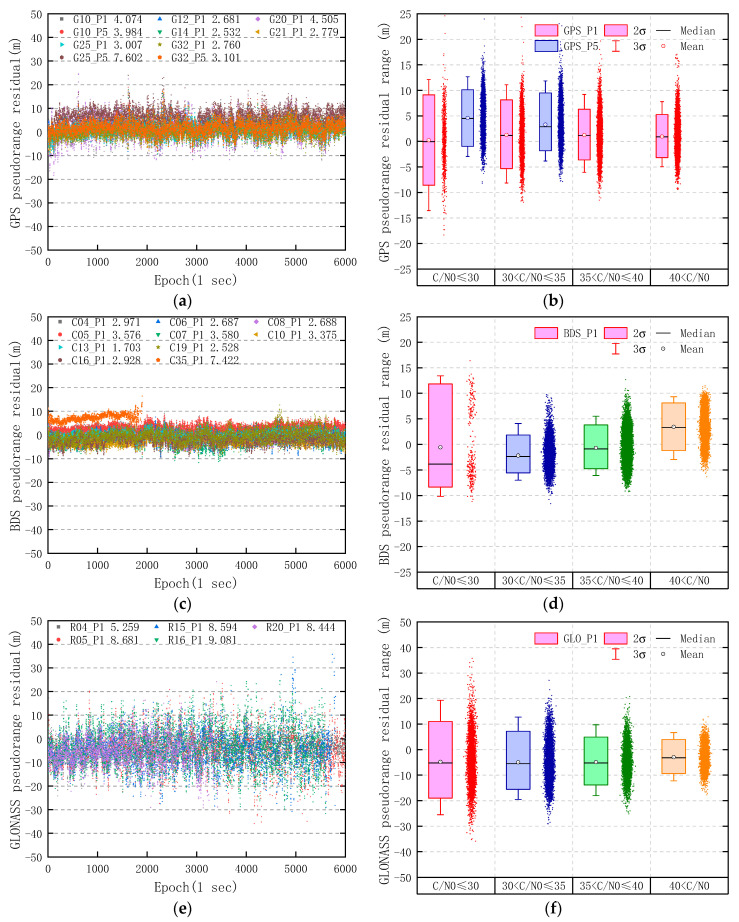
Pseudorange residuals of observed GPS, BDS, and GLONASS satellites in static data collected by the Huawei Mate30. (**a**) Pseudorange residual of GPS; (**b**) pseudorange residual range of GPS; (**c**) pseudorange residual of BDS; (**d**) pseudorange residual range of BDS; (**e**) pseudorange residual of GLONASS; and (**f**) pseudorange residual range of GLONASS. The value after the satellite pseudo random noise (PRN) number is the RMS of pseudorange residuals.

The raw code observation equation is written as:

(5) $$P_{i}=\rho +cdt_{r}-cdt^{s}+T+\frac{f_{1}^{2}}{f_{i}^{2}}I+B_{P_{i}}^{r}-B_{P_{i}}^{s}+\varepsilon_{P_{i}}$$

where ρ is the geometric distance between satellite and receiver, c is the speed of light in vacuum, $cdt_{r}$ is the receiver clock error, $cdt^{s}$ is the satellite clock error, T is the tropospheric delay, and the other symbols are the same as before. The coordinates of SYS2 were taken as known, and the products such as precise satellite orbit and clock, final ionospheric TEC grid, etc., were provided by the IGS data center. The effects of earth tide, relativity, and Sagnac are modeled and corrected sufficiently, and the code hardware delay is ignored. Moreover, the pseudorange residuals of GNSS satellites are computed with the prior information, and the results are not affected by the parameter estimation based on Kalman filter. Therefore, the receiver clock error is computed by least squares estimation in single point positioning (SPP). The pseudorange residuals of GPS, BDS, and GLONASS are shown in Figure 5, and it should be noted that $\varepsilon_{P_{i}}$ contains the multipath error.

In Figure 5, the statistical results show that the RMS value of pseudorange residuals of GPS, BDS, and GLONASS are 3.942, 3.196, and 8.063 m, respectively. Liu et al. [17] computed the RMS of GPS, BDS, and GLONASS pseudorange residuals at approximately 6–7, 3.09, and 12–13 m, with single difference (SD) for the Google Nexus9, Huawei P10, and Samsung S8, while the pseudorange error of surveying receiver was ignored. For most smartphones, the RMS of GLONASS pseudorange residuals may be lower than that of GPS and BDS. Moreover, with the increase of *C/N*_0_, the two-sigma (95% confidence level) range of GPS P1 pseudorange residual decreases from the initial 17.727 m (*C/N*_0_ ≤ 30) to 8.428 m (40 < *C/N*_0_); the range of BDS decreases from 20.157 m (*C/N*_0_ ≤ 30) to 9.341 m (40 < *C/N*_0_) and the range of GLONASS decreases from 30.105 m (*C/N*_0_ ≤ 30) to 13.314 m (40 < *C/N*_0_). Meanwhile, the *C/N*_0_ value of the GPS L5 band is generally lower than that of L1 band. There are only 11 GPS L5 observations with a value of *C/N*_0_ above 35 dB-Hz among the 100 min, which are so few that there is no need to draw them in Figure 5b. The average *C/N*_0_ values of L1 band of GPS PRN 10, PRN 25, and PRN 32 are 8.59, 12.18, and 9.46 dB-Hz higher than that of L5, respectively.

The RMS of GPS, BDS, and GLONASS pseudorange residuals with each *C/N*_0_ range is shown in Table 1. As the *C/N*_0_ value increased, the RMS of GPS, BDS, and GLONASS pseudorange residuals all decreased significantly. The number of GPS observations with a *C/N*_0_ value of L5 above 35 dB-Hz is so few that we did not include their statistics in Table 1. However, the RMS of BDS pseudorange residuals increases when the *C/N*_0_ value of BDS is above 40 dB-Hz, which is caused by the satellites with a low elevation, such as PRN 35.

## 3. Multi-GNSS PPP Mathematical Model

### 3.1. Uncombined Model

The mathematical models of PPP mainly include ionosphere-free combined [13,14] and uncombined. However, for most of the time, there are still not enough dual frequency satellites received by the Huawei Mate30 for ionosphere-free combined model even adding Galileo satellites. This is because only a few Galileo satellites such as PRN 33 observed by the Huawei Mate30 can be logged with dual frequency observations, and the most Galileo satellites are still logged with single frequency observations. In addition, for the uncombined PPP model based on SD between satellites, the GNSS satellite with the highest elevation angle is generally selected as the reference satellite index. Although the satellite with the highest *C/N*_0_ can be selected as the reference satellite index for smartphone positioning to ensure the quality of measurements of the reference satellite, the reference satellite may not have the L5 observations. Either the GPS L5 band observations are not used or the satellite with L5 frequency band is directly selected as the reference satellite index. Considering the scarcity of GPS L5 satellites and the advantage of dual frequency observations, the undifferenced and uncombined PPP model was used in this experiment. The multi-GNSS PPP model can be written as:

(6) $$P_{i}^{Q}=\rho^{Q}+cdt_{r}+cdt_{D}^{Q}-cdt^{Q}+T^{Q}+\frac{f_{1}^{2}}{f_{i}^{2}}I^{Q}+B_{P_{i}}^{r,Q}-B_{P_{i}}^{Q}+\varepsilon_{{}_{P_{i}}}^{Q}$$

(7) $$L_{i}^{Q}=\rho^{Q}+cdt_{r}+cdt_{D}^{Q}-cdt^{Q}+T^{Q}-\frac{f_{1}^{2}}{f_{i}^{2}}I^{Q}-\lambda_{i}^{Q}N_{i}^{Q}+B_{L_{i}}^{r,Q}-B_{L_{i}}^{Q}+\varepsilon_{L_{i}}^{Q}$$

where the superscript Q denotes the satellite system, $cdt_{D}^{Q}$ is time bias between Q satellite system and GPS, and the other symbols are the same as before. By Equations (6) and (7), the error equation of multi-GNSS observation can be written as:

(8) V=H⋅X+l

where the V is GNSS observation residual, H is the coefficient matrix of X, and the basic parameters can be expressed as:

(9) $$X=\left[\begin{array}{cccccccccccc} x, & y, & z, & cdt_{r}, & cdt_{D}^{B}, & cdt_{D}^{R}, & T, & {\hat{I}}_{1}, & {\hat{N}}_{1}^{G}, & {\hat{N}}_{5}^{G}, & {\hat{N}}_{1}^{B}, & {\hat{N}}_{1}^{R} \end{array}\right]$$

where the x, y, z are three-dimensional coordinates of the receiver, $cdt_{D}^{B}$ is BDS system time bias, $cdt_{D}^{R}$ is GLONASS system time bias, ${\hat{I}}_{1}$ is the ionospheric delay of L1, ${\hat{N}}_{1}^{G}$, ${\hat{N}}_{5}^{G}$, ${\hat{N}}_{1}^{B}$, and ${\hat{N}}_{1}^{R}$ are the integer ambiguity parameters of GPS, BDS, and GLONASS, respectively. The coefficient matrix H can be expressed as:

(10) $$H=\left[\begin{array}{cccccccccccccccccccc} {\hat{\partial }}_{1}^{G,1} & 1 & 0 & 0 & M_{W}^{1} & 1 & \cdots & 0 & 0 & \cdots & 0 & 0 & \cdots & 0 & 0 & \cdots & 0 & 0 & \cdots & 0\\ {\hat{\partial }}_{1}^{G,1} & 1 & 0 & 0 & M_{W}^{1} & -1 & \cdots & 0 & 1 & \cdots & 0 & 0 & \cdots & 0 & 0 & \cdots & 0 & 0 & \cdots & 0\\ {\hat{\partial }}_{1}^{G,5} & 1 & 0 & 0 & M_{W}^{1} & \mu_{5} & \cdots & 0 & 0 & \cdots & 0 & 0 & \cdots & 0 & 0 & \cdots & 0 & 0 & \cdots & 0\\ {\hat{\partial }}_{1}^{G,5} & 1 & 0 & 0 & M_{W}^{1} & -\mu_{5} & \cdots & 0 & 0 & \cdots & 0 & 1 & \cdots & 0 & 0 & \cdots & 0 & 0 & \cdots & 0\\ \vdots & \vdots & \vdots & \vdots & \vdots & \vdots & \ddots & \vdots & \vdots & \ddots & \vdots & \vdots & \ddots & \vdots & \vdots & \vdots & \vdots & \vdots & \vdots & \vdots \\ {\hat{\partial }}_{k}^{G,1} & 1 & 0 & 0 & M_{W}^{k} & 0 & \cdots & 1 & 0 & \cdots & 0 & 0 & \cdots & 0 & 0 & \cdots & 0 & 0 & \cdots & 0\\ {\hat{\partial }}_{k}^{G,1} & 1 & 0 & 0 & M_{W}^{k} & 0 & \cdots & -1 & 0 & \cdots & 1 & 0 & \cdots & 0 & 0 & \cdots & 0 & 0 & \cdots & 0\\ \vdots & \vdots & \vdots & \vdots & \vdots & \vdots & \ddots & \vdots & \vdots & \vdots & \vdots & \vdots & \vdots & \vdots & \vdots & \ddots & \vdots & \vdots & \vdots & \vdots \\ {\hat{\partial }}_{m}^{B,1} & 1 & 1 & 0 & M_{W}^{m} & 0 & \cdots & 1 & 0 & \cdots & 0 & 0 & \cdots & 0 & 0 & \cdots & 0 & 0 & \cdots & 0\\ {\hat{\partial }}_{m}^{B,1} & 1 & 1 & 0 & M_{W}^{m} & 0 & \cdots & -1 & 0 & \cdots & 0 & 0 & \cdots & 0 & 0 & \cdots & 1 & 0 & \cdots & 0\\ \vdots & \vdots & \vdots & \vdots & \vdots & \vdots & \ddots & \vdots & \vdots & \vdots & \vdots & \vdots & \vdots & \vdots & \vdots & \vdots & \vdots & \vdots & \ddots & \vdots \\ {\hat{\partial }}_{n}^{R,1} & 1 & 0 & 1 & M_{W}^{n} & 0 & \cdots & 1 & 0 & \cdots & 0 & 0 & \cdots & 0 & 0 & \cdots & 0 & 0 & \cdots & 0\\ {\hat{\partial }}_{n}^{R,1} & 1 & 0 & 1 & M_{W}^{n} & 0 & \cdots & -1 & 0 & \cdots & 0 & 0 & \cdots & 0 & 0 & \cdots & 0 & 0 & \cdots & 1 \end{array}\right]$$

where the ${\hat{\partial }}_{n}^{Q,i}=\left[\begin{array}{ccc} a^{n}, & b^{n}, & c^{n} \end{array}\right]$ is the line-of-sight vector between receiver and satellite, $M_{W}^{n}$ is the tropospheric projection coefficient with global mapping function (GMF) [23], $\mu_{i}=f_{1}^{2}/f_{i}^{2}$, and $f_{i}$ is the frequency.

### 3.2. C/N*_0_*-Dependent Stochastic Model

In high-precision positioning using a geodetic receiver, the stochastic model depending on elevation is commonly used, such as:

(11) $$\sigma_{S}^{2}=\sigma_{0}^{2}\csc\left(E\right)$$

where $\sigma_{0}^{2}$ is the precision of the observation at zenith and E is the elevation angle. However, according to the experiments of GNSS signal characteristics of smartphones, it is found that the *C/N*_0_-dependent weighting stochastic model is more suitable for GNSS positioning using Android smartphones [8,17]. Banville et al. [24] also indicated that carrier-to-noise weighting should replace elevation-dependent weighting, and proposed a measurement weighting based on carrier-to-noise ratio values suitable for processing of low-cost GNSS receiver data. Ward [25] derived a formula that expresses the phase variance $\sigma_{S}^{2}$ in ${\mathrm{mm}}^{2}$ as a function of the measured *C/N*_0_ values:

(12) $$\sigma_{S}^{2}=C_{S}\cdot 10^{\frac{-(C/N_{0}measured)}{10}}$$

and

(13) $$C_{S}=B_{S}{\left[\frac{\lambda }{2\pi }\right]}^{2}$$

where $B_{S}$ is the carrier tracking loop bandwidth (Hz), and the effect of the oscillator stability on the phase variances is considered negligible. Equation (12) can be used to estimate variances of the raw phase observations at one station to a satellite, i.e., undifferenced [26,27]. However, the constant value obtained by the fitting with the phase variances and *C/N*_0_ variances is usually used as the parameter $C_{S}$ due to the lack of bandwidth information. Meanwhile, taking into account the GNSS signal characteristics of smartphones, the method of segmental weighting is adopted by referring to the elevation-dependent weighting [28]. The step function variance can be given as:

(14) $$\sigma_{S}^{2}(C/N_{0})=\{\begin{array}{ll} C_{S}\cdot 10^{\frac{-(C/N_{0}measured)}{10}} & C/N_{0}>\alpha \\ C_{S}\cdot 10^{\frac{-(\kappa \cdot C/N_{0}measured)}{10}} & C/N_{0}\leq \alpha ,\kappa \in \left(0,1\right) \end{array}$$

where α is the threshold value of *C/N*_0_, κ is the coefficient of *C/N*_0_; the specific value can be determined by the gross measurement noise of satellites below the threshold value. Although some studies suggest that the cutoff value of *C/N*_0_ should be directly set, Liu et al. [17] suggested that the pseudorange observations whose *C/N*_0_ are below 30 dB-Hz should be excluded in the Android GNSS positioning process, and Guo et al. [29] indicated that GNSS observations with *C/N*_0_ less than 30 dB-Hz should be rejected. However, for some early smartphones, such as the Huawei Mate9, about 44.17% of the GNSS observations with the *C/N*_0_ below 30 dB-Hz in one hour, and the number of visible satellites that smartphones receive in urban environments is limited. Therefore, the cutoff *C/N*_0_ value is not directly set in the PPP mathematical model.

### 3.3. Parameter Estimation Model Based on Robust Kalman Filter

The Kalman filter is the most commonly used method for parameter estimation in multi-GNSS positioning. The extended Kalman filter (EKF) is a nonlinear version of Kalman filtering; the dynamic and observation models can be expressed as:

(15) $$X_{k+1}=\Phi_{k+1,k}{\hat{X}}_{k}+w_{k}$$

(16) $$L_{k+1}={\tilde{L}}_{k}+H_{k+1}(X_{k}-{\tilde{X}}_{k})+v_{k}$$

where $X_{k+1}$ is state vector, $L_{k+1}$ is observation vector, $\Phi_{k+1,k}$ is state transition matrix, and the vectors $w_{k}$ and $v_{k}$ are zero mean Gaussian white sequences having zero cross-correlation with each other [30]:

(17) $$E[w_{k}w_{i}^{T}]=\{\begin{array}{c} Q_{k},i=k\\ 0,i\neq k \end{array};E[v_{k}v_{i}^{T}]=\{\begin{array}{c} R_{k},i=k\\ 0,i\neq k \end{array};E[w_{k}v_{i}^{T}]=0$$

where $Q_{k}$ is the process noise covariance matrix and $R_{k}$ is the measurement noise covariance matrix. With the GPS/BDS/GLONASS priori covariance:

(18) $$R_{k}=\sigma_{S}^{2}P_{S}^{-1}=\left[\begin{array}{ccc} \sigma_{G}^{2}P_{G}^{-1} & 0 & 0\\ 0 & \sigma_{B}^{2}P_{B}^{-1} & 0\\ 0 & 0 & \sigma_{R}^{2}P_{R}^{-1} \end{array}\right]$$

where the subscript S denote satellite system, the subscript G, B, and R denote GPS, BDS, and GLONASS, respectively, $\sigma_{S}^{2}$ is the measurement error covariance, and $P_{S}$ is the weight matrix. Although it has been very common to add fictitious process noise to the system model, the best cure for nonconvergence caused by unmodeled states is to correct the model [30].

To improve the stability of PPP using a smartphone, the robust Kalman filter method is used for parameter estimation, which reduces the effects of observation outliers on positioning accuracy. According to the prior weight elements of the observation vector and the robust M estimation principle, the equivalent covariance matrix of observations error ${\bar{R}}_{k}$ instead of the original covariance $R_{k}$ for the robust Kalman filter, and the formula of robust Kalman gain matrix ${\bar{K}}_{k}$ follows [31]:

(19) $${\bar{K}}_{k}=P_{k}^{-}H_{k}^{T}{\left(H_{k}P_{k}^{-}H_{k}^{T}+{\bar{R}}_{k}\right)}^{-1}$$

where $P_{k}^{-}$ is the state covariance matrix and $H_{k}^{T}$ is the coefficient matrix. The equivalent covariance matrix ${\bar{R}}_{k}$ can be calculated as [32]:

(20) $${\bar{R}}_{k}=R_{k}/\gamma_{i}$$

where $\gamma_{i}$ is the variance inflation factor, which can be used to adjust the variance of observations by the institute of geodesy and geophysics (IGG) III weighting function [33].

(21) $$\gamma_{i}=\{\begin{array}{ll} 1 & \left|{\bar{v}}_{i}\right|\leq k_{0}\\ \frac{k_{0}}{\left|{\bar{v}}_{i}\right|}{\left(\frac{k_{1}-\left|{\bar{v}}_{i}\right|}{k_{1}-k_{0}}\right)}^{2} & k_{0}<\left|{\bar{v}}_{i}\right|\leq k_{1}\\ 0 & \left|{\bar{v}}_{i}\right|>k_{1} \end{array}$$

where $k_{0}$ and $k_{1}$ are two thresholds, usually chosen as 1.5–3.0 and 3.0–8.0, respectively; ${\bar{v}}_{i}$ is the standardized residual, which is defined as [34]:

(22) $${\bar{v}}_{i}=\frac{v_{i}}{\sqrt{{\hat{\sigma }}_{0}^{2}Q_{vi}}}$$

where $v_{i}$ is the observations residual and $Q_{vi}$ is the corresponding variance. ${\hat{\sigma }}_{0}^{2}$ is the estimate of unit weight variance, which can be calculated with generalized least squares principle:

(23) $${\hat{\sigma }}_{0}^{2}=\frac{\xi^{T}Q_{\xi }^{-1}\xi }{n}$$

where ξ is predicted residual vector (innovations) and n is the number of observations; $Q_{\xi }$ is the corresponding covariance matrix, which can be calculated as follows:

(24) $$Q_{\xi }=R_{k}+H_{k}P_{k}^{-}H_{k}^{T}$$

The update state correction vector and error covariance are:

(25) $${\hat{X}}_{k}={\hat{X}}_{\bar{k}}+{\bar{K}}_{k}(L_{k}-H_{k}{\hat{X}}_{\bar{k}})$$

(26) $$P_{k}=[I-{\bar{K}}_{k}H_{k}]P_{k}^{-}{\left[I-{\bar{K}}_{k}H_{k}\right]}^{T}+{\bar{K}}_{k}{\bar{R}}_{k}{\bar{K}}_{k}^{T}$$

## 4. Experiment and Result

The multi-GNSS positioning experiments including static and kinematic PPP were carried out with 100 min GPS, BDS, and GLONASS observations of the Huawei Mate30, and the GNSS observation bands include L1/L5 of GPS, B1 of BDS, and G1 of GLONASS. The products such as precise satellite orbit and clock, final GIM, antenna phase center correction, and differential code bias (DCB) were provided by the IGS data center [35]. The hydrostatic troposphere error was corrected with the Saastamoinen model [36] and the zenith delay of the wet troposphere was estimated as a parameter. The cutoff elevation of satellites was 15 degrees, and the ocean tide model was FES2004. The effects of earth tide, relativity, and Sagnac were modeled and corrected sufficiently. For comparison, the proposed PPP method adopted the *C/N*_0_-dependent stochastic model and robust Kalman filter parameter estimation model, while the elevation-dependent weighting method and standard Kalman filter were used in the conventional PPP method. Moreover, the integer ambiguities of carrier phase were all estimated as a float solution.

### 4.1. Multi-GNSS Static PPP Solution

**Figure 6:**
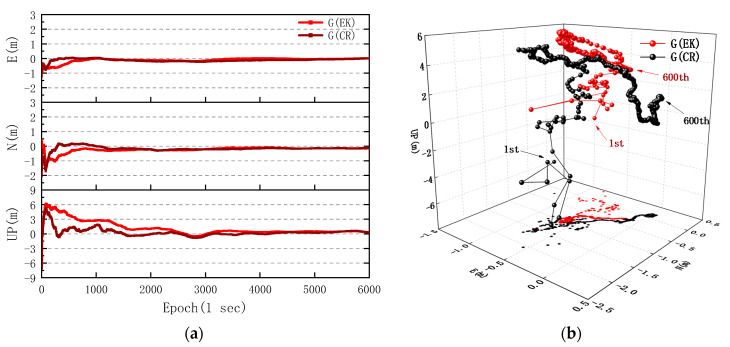

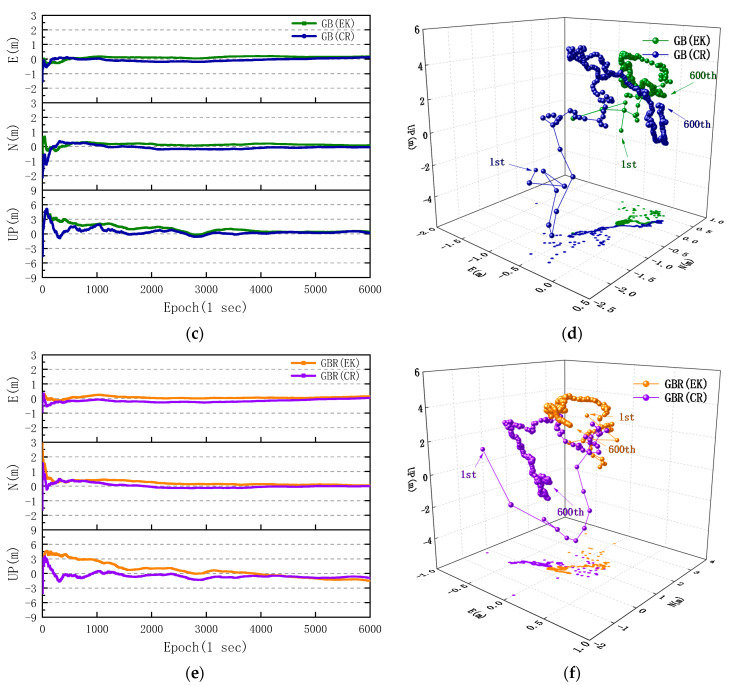
Comparison of the static PPP errors with different combinations of GPS, GPS/BDS, and GPS/BDS/GLONASS observed by the Huawei Mate30 in three directions. (**a**) GPS; (**b**) three-dimensional deviation points of GPS. (**c**) GPS/BDS; (**d**) three-dimensional deviation points of GPS/BDS. (**e**) GPS/BDS/GLONASS; (**f**) three-dimensional deviation points of GPS/BDS/GLONASS. The E and N denote east and north direction. The EK denotes the conventional PPP method with an elevation-dependent stochastic model and Kalman filter estimation model, and the CR denotes the proposed PPP method with a *C/N*_0_-dependent stochastic model and robust Kalman filter estimation model.

In view of the differences in measurement noise of GPS, BDS, and GLONASS observations of smartphone, the $C_{S}$ values of the *C/N*_0_-dependent stochastic model with step function variance of GPS, BDS, and GLONASS were calculated separately based on the previous experimental data of multi-GNSS pseudorange and carrier phase variance of the Huawei Mate30. Meanwhile, the appropriate weight matrix $P_{S}$ was set according to the measurement residuals of multi-GNSS observations and the orbit of satellites. Tests on the static PPP algorithms with elevation- and *C/N*_0_-dependent stochastic models were conducted with respect to different combinations of GPS, BDS, and GLONASS. It should be noted that the multi-GNSS observations of the Huawei Mate30 were collected in an open sky environment, which is less affected by multipath. The positioning errors of GPS, GPS/BDS, and GPS/BDS/GLONASS are shown in Figure 6a,c,e in terms of east, north and up directions of the coordinate system, and the corresponding three-dimensional deviation points in the initial 10 min are shown in Figure 6b,d,f.

According to the comparison of GNSS(CR) and GNSS(EK) in Figure 6b,d,f, it can be found that the positioning errors of GNSS(CR) are even higher than GNSS(EK) in the first few epochs, but the convergence rate of GNSS(CR) is obviously faster than that of GNSS(EK) with time in Figure 6a,c,e. In Figure 6a, the first time for the horizontal error of GPS(CR) convergence to 0.5 m is 156 epochs, which is much faster than 516 epochs of GPS(EK). The RMS of vertical error decreases from 1.850 m of GPS(EK) to 0.878 m of GPS(CR). The mean horizontal and vertical errors of GPS(CR) reach 0.234 and 0.502 m, respectively, which are lower than 0.267 and 1.205 m of GPS(EK). For the GPS/BDS and GPS/BDS/GLONASS positioning in Figure 6c,e, it should be emphasized that we had to weaken the allocation of carrier phase residuals correction of BDS in both of PPP methods, so as to improve the stability and accuracy of multi-GNSS positioning. The maximum vertical error decreases from 6.204 m for GPS(EK) to 4.607 m for GPS/BDS(EK) and 4.656 m for GPS/BDS/GLONASS(EK). Moreover, the RMS of vertical errors decreases from 1.333 m for GPS/BDS(EK) and 1.545 m for GPS/BDS/GLONASS(EK) to 0.817 m for GPS/BDS(CR) and 0.761 m for GPS/BDS/GLONASS(CR), respectively.

**Figure 7:**
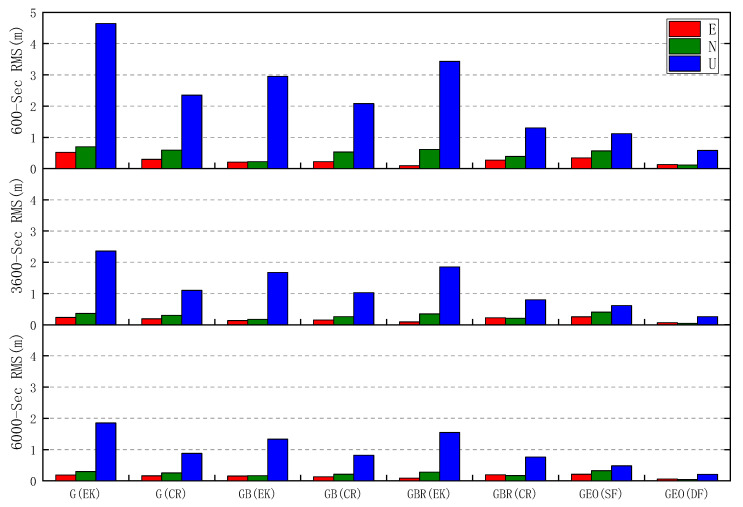
Comparison of the RMS of static PPP errors with respect to GPS, GPS/BDS, and GPS/BDS/GLONASS observed by the Huawei Mate30 in 10, 60, and 100 min. The RMS of single frequency and dual frequency PPP errors of the geodetic receiver during the same time period is presented for comparison. GEO denotes geodetic receiver; SF and DF denote single frequency and dual frequency, respectively.

The comparison of RMS of GPS, GPS/BDS, and GPS/BDS/GLONASS positioning errors in each direction during different periods is shown in Figure 7, and the RMS of single and dual frequency PPP errors of the geodetic receiver are added for reference. In the first 10 min, the RMS of three-dimensional positioning errors of GPS(CR), GPS/BDS(CR), and GPS/BDS/GLONASS(CR) are 2.444, 2.160 and 1.386 m respectively, which are much lower than 4.726, 2.969 and 3.488 m for GPS(EK), GPS/BDS(EK), and GPS/BDS/GLONASS(EK). Meanwhile, the RMS of horizontal and vertical errors of GPS/BDS/GLONASS(CR) is 0.474 and 1.302 m, respectively, which are close to 0.661 and 1.119 m for GEO(SF). Then the RMS of GPS/BDS/GLONASS(CR) positioning errors gradually decreases with time, of which the horizontal and vertical parts decrease continuously from 0.306 and 0.798 m within 60 min to 0.250 and 0.761 m within 100 min, respectively. However, the three-dimensional positioning errors of the proposed static PPP algorithm based on *C/N*_0_-dependent step function variance stochastic model decrease by different levels, no matter which group of GPS, GPS/BDS, and GPS/BDS/GLONASS is considered.

The RMS values of static PPP errors with respect to GPS, GPS/BDS, and GPS/BDS/GLONASS observed by the Huawei Mate30 and the geodetic receiver are shown in Table 2. Compared with GPS(EK), GPS/BDS(EK), and GPS/BDS/GLONASS(EK), the RMS of the three-dimensional positioning errors of GPS(CR), GPS/BDS(CR), and GPS/BDS/GLONASS(CR) decrease by 50.72%, 36.95%, and 49.06% to 0.928, 0.852, and 0.801 m in 100 min. Meanwhile, taking positioning based on GPS/BDS/GLONASS as an example, it can be seen from the Table 2 that the horizontal positioning accuracy of the proposed PPP algorithm for the smartphone has reached the level of single frequency PPP using a geodetic receiver, while the vertical one is relatively lower. It should be further noted that it takes less time for PPP positioning using the Huawei Mate30 to meet different accuracy levels of 0.5, 0.2, and 0. 1m, compared with single frequency PPP positioning using the geodetic receiver. In addition, for the PPP positioning using the Huawei Mate30, 329, 3874, and 4833 epochs are the cost, whereas for the single frequency PPP positioning using the geodetic receiver, 1426, 4031, and 5730 epochs are the cost. However, it can be seen from Table 2 that there is still a large gap in terms of the precision of multi-GNSS dual frequency PPP between the Huawei Mate30 and geodetic receiver.

### 4.2. Multi-GNSS Kinematic PPP Solution

**Figure 8:**
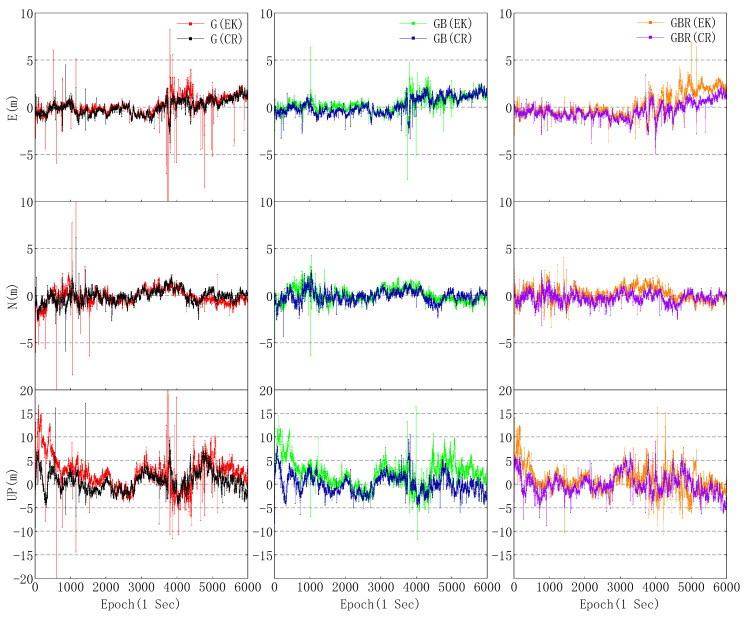
Comparison of the kinematic PPP errors between the conventional and the proposed models with respect to three combinations of GPS, BDS, and GLONASS.

The same set of GNSS data observed by the Huawei Mate30 was used for kinematic positioning with the two PPP methods described above. Unlike the static PPP method, the receiver position and corresponding error covariance should be initialized per epoch in the process of kinematic PPP, and the receiver position initialized from SPP which is estimated using the pseudorange on the L1 frequency with a variance of 60^2^ (m^2^). Moreover, it should be noted that the receiver clock should be initialized with a variance of 60^2^ (m^2^) per epoch both in static and kinematic PPP, and other parameters such as ambiguity are usually initialized at the beginning, which is the same as static PPP. The kinematic positioning biases of different combinations of GPS, BDS, and GLONASS in three directions are shown in Figure 8.

From Figure 8, although the maximum error of GPS in three directions is significantly reduced, it can be observed that there are still some large variations in GPS(CR), and the maximum three-dimensional error of GPS(CR) still reaches 17.442 m. The reason for this may be attributed to the residuals of many satellites, which are outliers and difficult to eliminate in the process of iteration. Therefore, with validity of sufficient observations, the maximum three-dimensional errors of GPS/BDS(CR) and GPS/BDS/GLONASS(CR) reduce to 11.083 and 10.317 m, respectively. Moreover, it can be seen that there is no obviously convergent pattern in the kinematic PPP error series in Figure 8, which is different from that of the geodetic receiver. It may be caused by the high code noise and limited to terms of both quality and quantity of L5 observations, which further affect the convergence of kinematic PPP.

Compared with the positioning error between GPS(EK) and GPS(CR), the RMS of GPS(CR) kinematic PPP error in east and north direction is similar to that of GPS(EK), but the proposed algorithm still outperforms the conventional one in two aspects, one is that the horizontal maximum error was reduced from 20.899 m for GPS(EK) to 7.422 m for GPS(CR) and the other is that RMS of vertical positioning error decreases from 4.162 m for GPS(EK) to 2.235 m for GPS(CR). Meanwhile, it can be observed that there is a significant reduction in maximum error of horizontal and vertical for GPS/BDS and GPS/BDS/GLONASS based on the proposed PPP method. As shown in Table 3, there is no significant difference in the RMS of horizontal errors between GPS/BDS(EK) and GPS/BDS(CR), but the horizontal maximum error decreases from 9.032 m for GPS/BDS(EK) to 4.973 m for GPS/BDS(CR), and the RMS of vertical errors decreases by 43.05% to 2.062 m. Compared with GPS/BDS/GLONASS(EK), the RMS of GPS/BDS/GLONASS(CR) horizontal and vertical error are reduced by 24.29% and 20.41% respectively. Moreover, the stability and reliability of multi-GNSS positioning using data from smartphones is still restricted by the poor multipath suppression capability and phase center deviation of the passive linearly polarized embedded GNSS antenna. Compared with the geodetic receiver, the multipath effects, noise level, and the number of observations gaps are much larger, which will affect the smartphone positioning discontinuities in kinematic PPP mode.

## 5. Conclusions and Remark

In this study, we conducted a comprehensive and detailed analysis of GNSS signal characteristics from the aspects of duty cycle, carrier-to-noise ratio, cycle slip, gradual accumulation of phase error, and pseudorange residual on a smartphone. The main conclusions are summarized as follows:

(1) The duty cycle can seriously affect the carrier phase observation data logging on some early smartphones, such as the Huawei Mate9, but it has little effect on some new smartphones such as the Huawei Mate30 and Huawei P30.
(2) Unlike the geodetic receiver, the GNSS satellites with high elevation do not necessarily bring the high carrier-to-noise ratio on the smartphone. The rate of GNSS carrier phase cycle slip on the Huawei Mate30 is inversely related to the carrier-to-noise ratio, and the most cycle slips are largely concentrated in the carrier-to-noise ratio below 30 dB-Hz. This means that the conventional stochastic model depending on elevation is difficult to accurately reflect the GNSS observation quality of the smartphone.
(3) In the phase–code differences experiment, the gradual accumulation of phase errors is most marked in BDS on the Huawei Mate30, and the trends correspond to a change of about −1.401 cm/s for BDS. Meanwhile, some extra cycle slips in BDS can be detected by MPF when the difference value between the carrier phase observations (in meters) and the pseudorange observations reached the threshold of 50 m. Moreover, the L5 band of GPS is also affected by the gradual accumulation of phase errors, but the trends are hard to draw.
(4) The comparison results of GNSS pseudorange residuals show that the RMS of GLONASS pseudorange residuals on the Huawei Mate30 is lower than that of GPS and BDS. Moreover, as the *C/N*_0_ value increased, the RMS of GPS, BDS, and GLONASS pseudorange residuals all decreased significantly.

In view of the GNSS signal characteristics of smartphones and to optimize the performance of multi-GNSS PPP, we propose a PPP mathematic method. The proposed undifferenced and uncombined multi-GNSS PPP model with single frequency and dual frequency observations is more suitable for these types of observations on smartphones. Unlike the geodetic receiver, there are significant differences between the gross measurement noises of GPS, BDS, and GLONASS observations from the smartphone. Therefore, we establish separately the *C/N*_0_-dependent stochastic model with step function variance according to the statistical data on variance of GPS, BDS, and GLONASS observations, and the appropriate weight matrix is set to improve the multi-GNSS positioning performance of smartphones. Moreover, the robust Kalman filter is used in parameter estimation, the equivalent variance matrix balances the contribution of the normal and abnormal observations. The experimental results show that the proposed PPP method converges more quickly than the conventional PPP method, depending on elevation under static conditions both the RMS and the maximum of multi-GNSS positioning errors decrease in kinematic tests. In addition, the horizontal positioning accuracy in GPS/BDS/GLONASS static PPP tests without significant signal obstructions and strong multipath reflectors using the Huawei Mate30 reached 10 cm within 4833 epochs, and it is even faster than the single frequency PPP using data from the geodetic receiver within 5730 epochs. However, the changing multipath and obstructions in urban environments can significantly affect the tracking of satellite signals in smartphones in kinematic conditions, which can lead to the decrease of the reliability of high-precision positioning for smartphones. Nevertheless, with the upgrade of the GNSS chip and antenna of smartphones, the adverse effects of GNSS signals characteristics such as gradual accumulation of phase errors, low carrier-to-noise ratio of the L5 band, high pseudorange residual range, and excessive rate of cycle slips in GLONASS, etc., will be overcome to some extent. By then, we can obtain high-precision and reliable location information using smartphones, even in urban streets where some satellite signals may be blocked.

## Figures and Tables

**Table 1:** Root mean square (RMS) of GPS, BDS, and GLONASS pseudorange residuals of the Huawei Mate30 in different *C/N*_0_ ranges.

*C/N*_0_ (dB-Hz)	Pseudorange Residuals RMS (m)			
	GPS		BDS	GLONASS
	P1	P5	P1	P1
*C/N*_0_ ≤ 30	5.388	5.730	6.964	10.604
30 < *C/N*_0_ ≤ 35	4.291	4.786	3.160	8.483
35 < *C/N*_0_ ≤ 40	3.307	N/A	2.762	7.497
40 < *C/N*_0_	2.774	N/A	4.320	5.032

**Table 2:** RMS of GPS, GPS/BDS, and GPS/BDS/GLONASS static PPP errors of the Huawei Mate30 and geodetic receiver. 3D denotes three-dimensional.

	Static Precise Point Positioning Error RMS (m)							
Direction	Huawei Mate30						Geodetic Receiver	
	G(EK)	G(CR)	GB(EK)	GB(CR)	GBR(EK)	GBR(CR)	SF	DF
10 min								
E	0.520	0.298	0.207	0.221	0.095	0.273	0.341	0.129
N	0.701	0.594	0.221	0.533	0.614	0.387	0.567	0.114
U	4.645	2.352	2.954	2.081	3.432	1.302	1.119	0.580
3D	4.726	2.444	2.969	2.160	3.488	1.386	1.300	0.605
100 min								
E	0.183	0.160	0.150	0.125	0.085	0.188	0.207	0.056
N	0.293	0.253	0.160	0.207	0.276	0.165	0.323	0.037
U	1.850	0.878	1.333	0.817	1.545	0.761	0.478	0.205
3D	1.882	0.928	1.351	0.852	1.572	0.801	0.613	0.216

**Table 3:** RMS of GPS, BDS, and GLONASS kinematic PPP errors in three directions within 100 min. MAX denotes maximum three-dimensional error.

Direction	Kinematic Precise Point Positioning Error RMS (m)					
	G(EK)	G(CR)	G + B(EK)	G + B(CR)	G + B + R(EK)	G + B +R(CR)
E	0.962	0.763	0.866	0.897	1.257	0.928
N	0.857	0.707	0.754	0.640	0.778	0.624
U	4.162	2.235	3.621	2.062	2.722	2.167
Max	35.991	17.442	17.298	11.083	16.543	10.317

## References

[1] Banville S., Diggelen F.V. (2016). Precise GNSS for Everyone: Precise Positioning Using Raw GPS Measurements from Android Smartphones. GPS World.

[2] Malkos S. (2016). Google to Provide Raw GNSS Measurements. GPS World.

[3] Paziewski J. (2020). Recent advances and perspectives for positioning and applications with smartphone GNSS observations. Meas. Sci. Technol..

[4] Humphreys T.E., Murrian M., van Diggelen F., Podshivalov S., Pesyna K.M. On the Feasibility of cm-Accurate Positioning via a Smartphone’s Antenna and GNSS Chip. Proceedings of the 2016 IEEE/Ion Position, Location and Navigation Symposium.

[5] Eugenio R., Stefano C., Lisa P., Daniele S. (2017). Precise GNSS Positioning Using Smart Devices. Sensors.

[6] Zhang X.H., Tao X.L., Zhu F., Shi X., Wang F.H. (2018). Quality assessment of GNSS observations from an Android N smartphone and positioning performance analysis using time-differenced filtering approach. GPS Solut..

[7] Li G.C., Geng J.H. (2019). Characteristics of raw multi-GNSS measurement error from Google Android smart devices. GPS Solut..

[8] Paziewski J., Sieradzki R., Baryla R. (2019). Signal characterization and assessment of code GNSS positioning with low-power consumption smartphones. GPS Solut..

[9] Wanninger L., Hesselbarth A. (2020). GNSS code and carrier phase observations of a Huawei P30 smartphone: Quality assessment and centimeter-accurate positioning. Gps Solut..

[10] Chen B., Gao C., Liu Y., Sun P. (2019). Real-time Precise Point Positioning with a Xiaomi MI 8 Android Smartphone. Sensors.

[11] Elmezayen A., El-Rabbany A. (2019). Precise Point Positioning Using World’s First Dual-Frequency GPS/GALILEO Smartphone. Sensors.

[12] Wu Q., Sun M.F., Zhou C.J., Zhang P. (2019). Precise Point Positioning Using Dual-Frequency GNSS Observations on Smartphone. Sensors.

[13] Zumberge J.F., Heflin M.B., Jefferson D.C., Watkins M.M., Webb F.H. (1997). Precise point positioning for the efficient and robust analysis of GPS data from large networks. J. Geophys. Res..

[14] Kouba J., Héroux P. (2001). Precise Point Positioning Using IGS Orbit and Clock Products. GPS Solut..

[15] Linty N., Lo Presti L., Dovis F., Crosta P. Performance analysis of duty-cycle power saving techniques in GNSS mass-market receivers. Proceedings of the 2014 Ieee/Ion Position, Location and Navigation Symposium—Plans 2014.

[16] Håkansson M. (2018). Characterization of GNSS observations from a Nexus 9 Android tablet. GPS Solut..

[17] Liu W.K., Shi X., Zhu F., Tao X.L., Wang F.H. (2019). Quality analysis of multi-GNSS raw observations and a velocity-aided positioning approach based on smartphones. Adv. Space Res..

[18] Lachapelle G., Gratton P. GNSS Precise Point Positioning with Android Smartphones and Comparison with High Performance Receivers. Proceedings of the 2019 IEEE International Conference on Signal, Information and Data Processing (ICSIDP).

[19] Gill M., Bisnath S., Aggrey J., Seepersad G., Inst N. Precise Point Positioning (PPP) using Low-Cost and Ultra-Low-Cost GNSS Receivers. Proceedings of the 30th International Technical Meeting of the Satellite Division of the Institute of Navigation.

[20] Zangeneh-Nejad F., Amiri-Simkooei A.R., Sharifi M.A., Asgari J. (2017). Cycle slip detection and repair of undifferenced single-frequency GPS carrier phase observations. GPS Solut..

[21] Zhao J., Hernández-Pajares M., Li Z., Wang L., Yuan H. (2020). High-rate Doppler-aided cycle slip detection and repair method for low-cost single-frequency receivers. GPS Solut..

[22] Li B.F., Liu T.X., Nie L.W., Qin Y.N. (2019). Single-frequency GNSS cycle slip estimation with positional polynomial constraint. J. Geod..

[23] Boehm J., Niell A., Tregoning P., Schuh H. (2006). Global Mapping Function (GMF): A new empirical mapping function based on numerical weather model data. Geophys. Res. Lett..

[24] Banville S., Lachapelle G., Ghoddousi-Fard R., Gratton P., Inst N. Automated Processing of Low-Cost GNSS Receiver Data. Proceedings of the 32nd International Technical Meeting of the Satellite Division of the Institute of Navigation.

[25] Ward P. (1996). Satellite signal acquisition and tracking. Understanding GPS: Principles and Applications.

[26] Brunner F.K., Hartinger H., Troyer L. (1999). GPS signal diffraction modelling: The stochastic SIGMA-δ model. J. Geod..

[27] Hartinger H., Brunner F.K. (1999). Variances of GPS Phase Observations: The SIGMA-ɛ Model. GPS Solut..

[28] Witchayangkoon B. (2000). Elements of GPS precise point positioning. Spatial Information Science and Engineering.

[29] Guo L., Wang F.H., Sang J.Z., Lin X.H., Gong X.W., Zhang W.W. (2020). Characteristics Analysis of Raw Multi-GNSS Measurement from Xiaomi Mi 8 and Positioning Performance Improvement with L5/E5 Frequency in an Urban Environment. Remote Sens..

[30] Jwo D.J., Lai C.N. (2008). Unscented Kalman filter with nonlinear dynamic process modeling for GPS navigation. Gps Solut..

[31] Yang Y., He H., Xu G. (2001). Adaptively robust filtering for kinematic geodetic positioning. J. Geod..

[32] Guo F., Zhang X.H. (2014). Adaptive robust Kalman filtering for precise point positioning. Meas. Sci. Technol..

[33] Yang Y., Song L., Xu T. (2002). Robust estimator for correlated observations based on bifactor equivalent weights. J. Geod..

[34] Zhang Q.Q., Zhao L.D., Zhao L., Zhou J.H. (2018). An Improved Robust Adaptive Kalman Filter for GNSS Precise Point Positioning. IEEE Sens. J..

[35] Kouba J. (2009). A Guide to Using International GNSS Service (IGS) Products.

[36] Saastamoinen J. (1972). Contributions to the theory of atmospheric refraction. Bull. Géodésique (1946–1975).

